# Electromagnetic Interference in Measurements of Radial Stress During Split Hopkinson Pressure Bar Experiments

**DOI:** 10.1007/s11340-017-0280-4

**Published:** 2017-04-03

**Authors:** A.D. Barr, S.D. Clarke, A. Tyas, J.A. Warren

**Affiliations:** 1 Department of Civil and Structural Engineering, The University of Sheffield, Mappin Street, Sheffield, S1 3JD UK; 2 Blastech Ltd., The BioIncubator, 40 Leavygreave Road, Sheffield, S3 7RD UK

**Keywords:** Split Hopkinson pressure bar, Radial stress, Electromagnetic interference, Soils

## Abstract

Split Hopkinson pressure bar experiments on soils are often carried out using a rigid steel confining ring to provide plane strain conditions, and measurements of the circumferential strain in the ring can be used to infer the radial stress on the surface of the specimen. Previous experiments have shown evidence of irregular electromagnetic interference in measurements of radial stress, which obscures the signals and impedes analysis. The development of robust constitutive models for soils in blast and impact events relies on the accurate characterisation of this behaviour, and so it is necessary to isolate and remove the source of interference. This paper uses an induction coil to identify the source of the anomalous signals, which are found to be due to induced currents in the gauge lead wires from the movement of magnetised pressure bars (martensitic stainless steel, 440C). Comparative experiments on sand and rubber specimens are used to show that the deforming soil specimen does not make a significant contribution to this activity, and recommendations are made on reducing electromagnetic interference to provide reliable radial stress measurements.

High-strain-rate testing of soils using the split Hopkinson pressure bar (SHPB) is typically driven by a need to understand the behaviour of soils in events such as buried explosions [1] and high-velocity fragment impact [2, 3]. As many soils are cohesionless, these experiments are often performed under uniaxial strain through use of a rigid confining ring (e.g. Fig. 1(a)), which constrains the lateral displacement of the specimen [4–9]. The addition of a circumferential strain gauge to the confining ring enables measurements of the radial stress in the specimen [1–3].

**Fig. 1:**
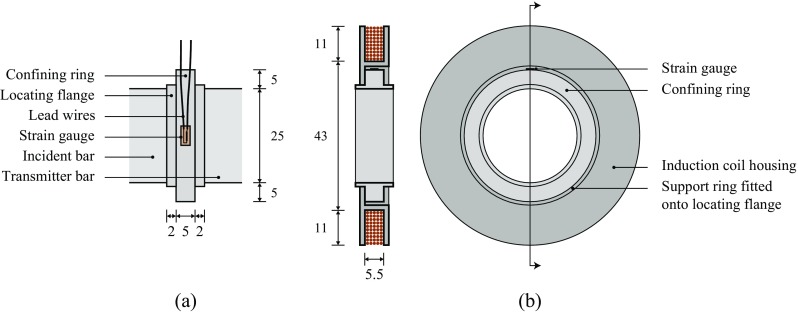
Arrangement of (**a**) gauged confining ring during a SHPB experiment (plan), (**b**) a 200-turn induction coil fitted to a confining ring (section and side view)

It was noted in previous experiments that the radial signals obtained from the confining ring were regularly affected by what appeared to be electrical noise [10]. This noise was most apparent at the beginning of the specimen loading, when stresses were low, and would appear as additional peaks or troughs in the radial stress signal. An example is shown in Fig. 2(a), where an additional trough obscures the signal at the beginning of the stress pulse, and appears to attenuate the remainder of the signal compared to the unaffected experiment in Fig. 2(b). This trough denotes a circumferential compression in the ring of 25 MPa, which is impossible under the applied loading, indicating that the source is electrical in nature. As experiments affected by the additional signals cannot be used to study the behaviour of the specimen, it is desirable to identify and remove the source of electromagnetic interference.

**Fig. 2:**
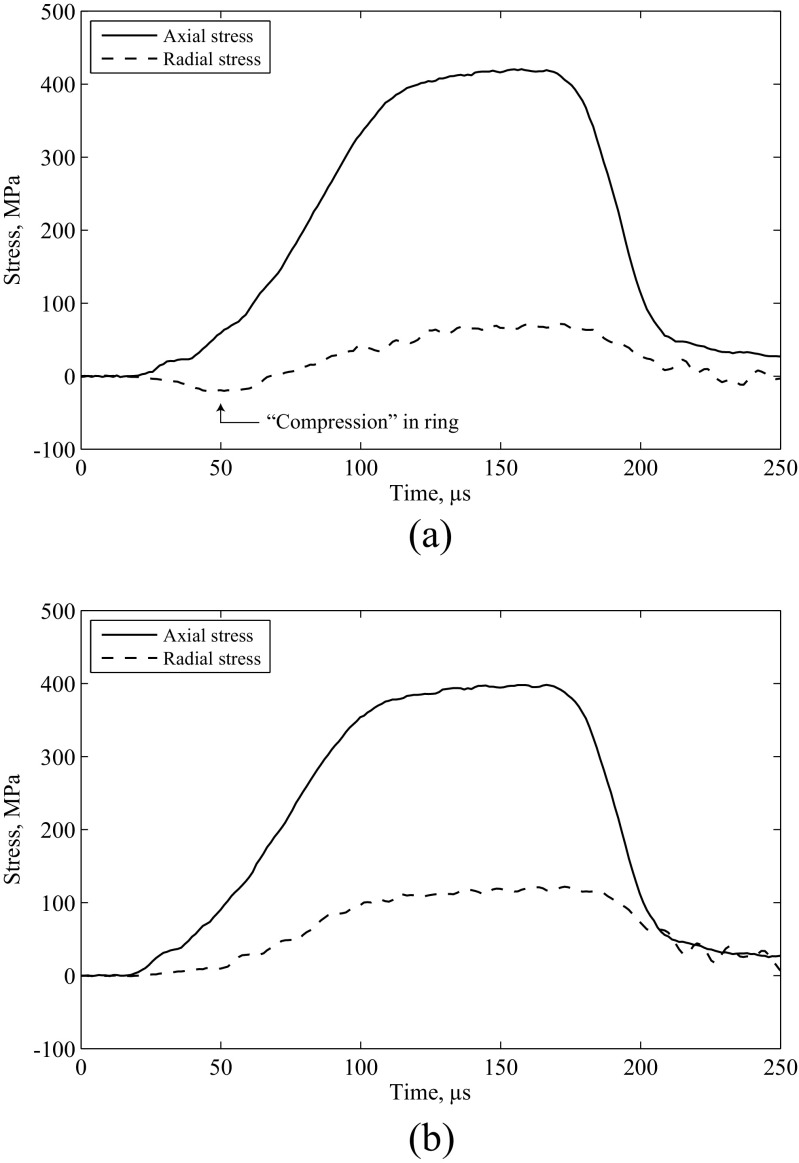
Examples of axial and radial stresses in confined SHPB experiments on sand, where (**a**) electromagnetic interference has introduced additional features in the radial stress signal, and (**b**) no interference was observed

Similar spurious signals were observed in transient pressure bar signals by Meitzler [11] and Vigness [12], and were identified as being due to magnetostrictive effects in the wire strain gauges. Magnetostriction is an effect observed in magnetic materials where a change in the magnetic state of the material results in a change in its length, due to the realignment of magnetic domains. The reciprocal Villari effect occurs when a change in the length of a magnetic material results in a change in its magnetic state, which can in turn induce a current in the material.

While magnetostrictive effects can account for fluctuations in the signal from ferromagnetic wire gauges, the semiconductor gauges used in the current experiments (Kyowa KSP-2-120-E4) use p-type silicon as the resistive element, where the change in resistance with strain is almost entirely due to the effect of piezoresistance rather than the change of geometry. However, the gauge, lead wires and other cables forming the strain gauge circuit remain susceptible to induced currents from simple electromagnetic induction, and the source of a fluctuating magnetic field in this case could be the pressure bars, confining ring or a nearby electrical source. Cress et al. [13] demonstrated that the fracture of many types of rock is accompanied by electrical activity due to the relative movement of charged surfaces, and so the fracture and movement of sand particles during the experiment may itself be a magnetic source.

In order to identify any sources of electromagnetic activity, and to understand how this may affect the radial stress measurements, an induction coil was designed to fit around the confining ring, as shown in Fig. 1(b). The coil consisted of 200 turns of 0.5 mm diameter single-core copper magnet wire arranged in ten-turn rows, and was secured using a polystyrene housing which was attached by interference fit to one of the confining ring flanges. A 2 mm clearance between the coil housing and the main body of the confining ring ensured that the coil was not strained during testing, so that any measurements could be confidently attributed to an electromagnetic source. Electromotive forces (EMFs) are generated in the coil according to Faraday’s law of induction

1 $$ \mathcal{E} = - N \frac{\!\mathrm{d} (BA)}{\!\mathrm{d} t} $$

where $\mathcal {E}$ is the EMF, *N* is the number of turns in the coil, *B* is the external magnetic field, and *A* is the area of the coil perpendicular to the field [14].

To isolate the potential contributions of the specimen and pressure bars, experiments using the confining ring and induction coil were first carried out with only a 5 mm air gap between two 1500 mm long 440C stainless steel pressure bars. An incident wave was applied using a 350 mm striker bar fired from a gas gun, and measurements of pressure bar strain and induction coil voltage were recorded using Wheatstone bridges connected to a TiePie Handyscope four-channel digital oscilloscope, with samples taken at 14-bit A-D resolution and at a sample rate of 1.562 MHz. Oscillations in the incident waves shown in Figs. 3 and 5 are due to dispersion of the stress wave as it propagates in the bar. A dispersion-correction technique was used to more accurately represent the dispersed signal at the bar–specimen interface [15].

**Fig. 3:**
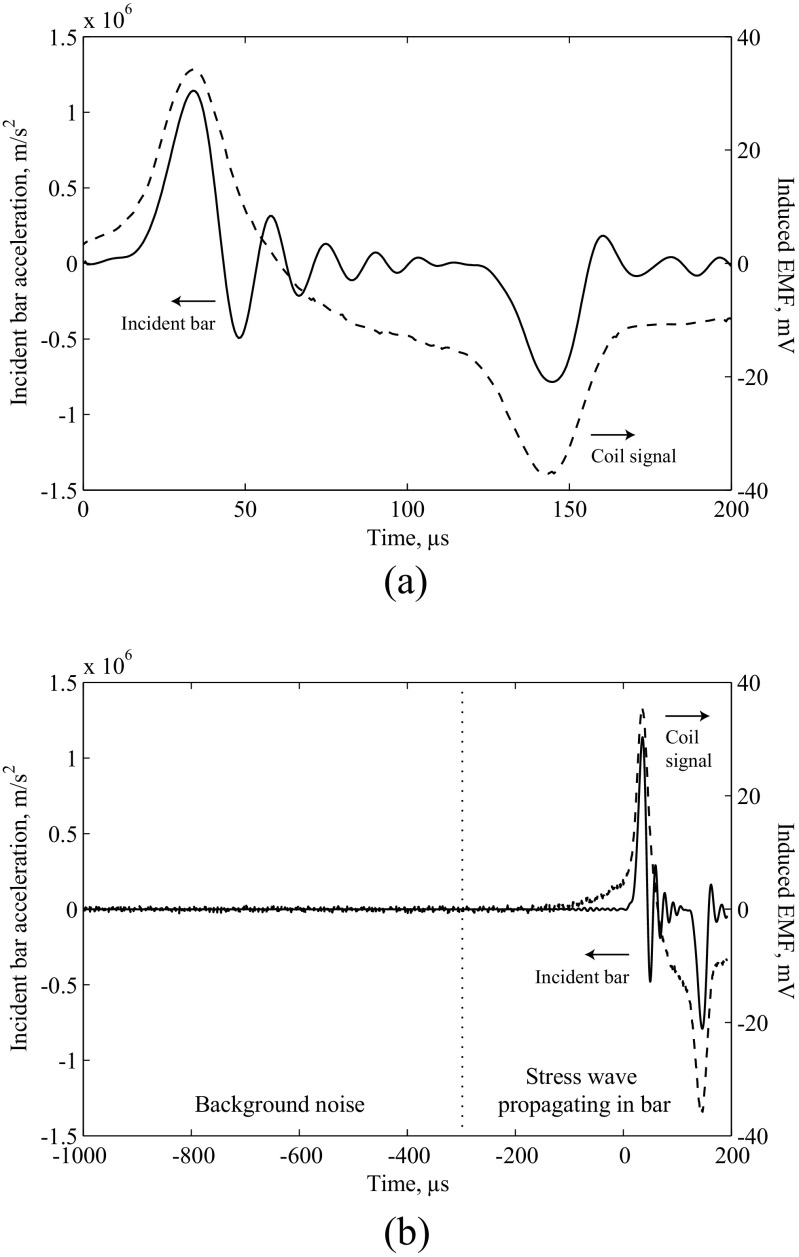
(**a**) Relationship between acceleration of the incident bar at the bar-specimen interface and the induction coil signal in a SHPB experiment with a 5 mm air specimen. Acceleration positive towards the coil. (**b**) Time base expanded to show extent of background noise

An EMF was induced in the coil as shown in Fig. 3(a), and appears to be associated with the acceleration of the incident bar: the transmitter bar remains stationary over the period of interest due to the air gap. The expanded time base in Fig. 3(b) shows that background noise from other sources exists in the circuit before the experiment is initiated and stress waves are propagating in the incident bar; however, this noise is several orders of magnitude smaller than the EMF generated during the bar’s acceleration. If a constant magnetic field existed around the incident bar the EMF in the coil would be expected to be proportional to the bar velocity: the correlation with acceleration implies that the strength of the magnetic field is also changing. Such a variation of the magnetic field with velocity (which is proportional to the stress in the bar) resembles the Villari effect, suggesting that the transient stresses in the bar lead to transient magnetisation.

As described by equation (1), EMFs are generated by changes in the magnetic field which passes through the area enclosed by the coil or wire loop, which in the case of the coil is in a well-defined plane parallel to the bar cross-section, as shown in Fig. 4(a). In contrast, the loop formed by the gauge lead wires is nominally tangential to the surface of the confining ring, and varies in position from test to test due to the thin, flexible wire. The area of the loop perpendicular to the magnetic field therefore varies considerably depending on the relative position of the two lead wires in any particular experiment, as demonstrated in Fig. 4(b). Where a large area cuts the field a larger EMF will be generated, and where a small area cuts the field a smaller EMF will be generated, leading to variation in the interference observed from test to test. The generation of peaks or troughs in each case will depend on the polarity of the strain gauge circuit during a particular test.

**Fig. 4:**
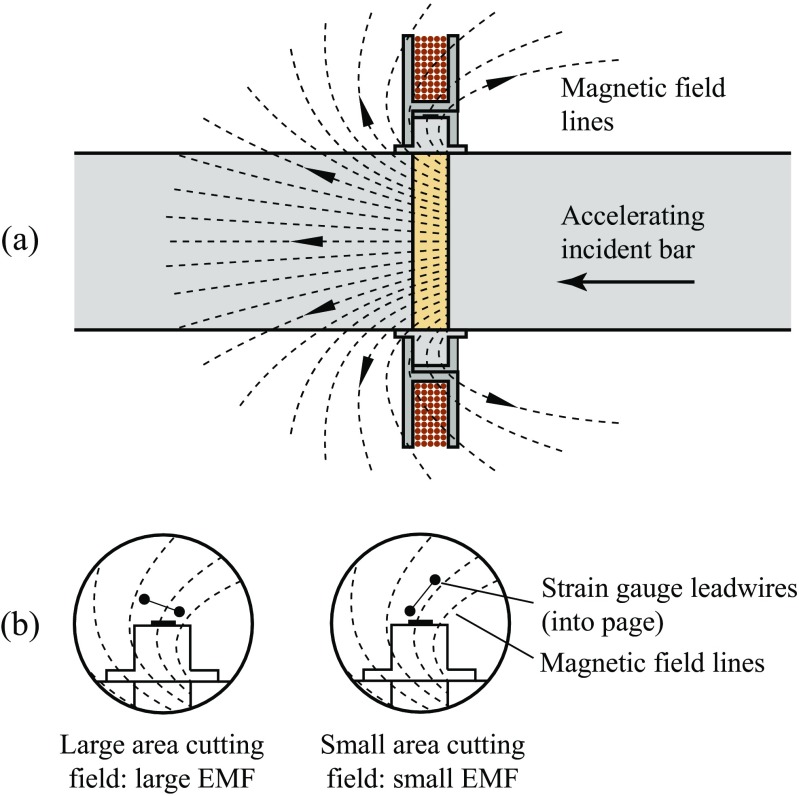
Diagrammatic example of the field around a magnetised incident bar in relation to (**a**) the induction coil, and (**b**) the lead wires on the confining ring strain gauge

To investigate the potential contribution of sand particle breakage as a source of electromagnetic activity, experiments on 5 mm long specimens of dry fine and medium sand (BS EN ISO 14688-1:2002) and natural rubber were also carried out using the confining ring and induction coil. In both cases the induction coil signals were similar to those in Fig. 3, and a number of experiments showed EMF interference in the radial strain measurements. This is shown in Fig. 5, where the more obvious additional peaks (▴) are highlighted: further interference may be present but cannot be reliably distinguished from the strain gauge signal. As a similar effect is observed in both sand and rubber specimens, the contribution from particle breakage in the sand does not appear to be significant.

**Fig. 5:**
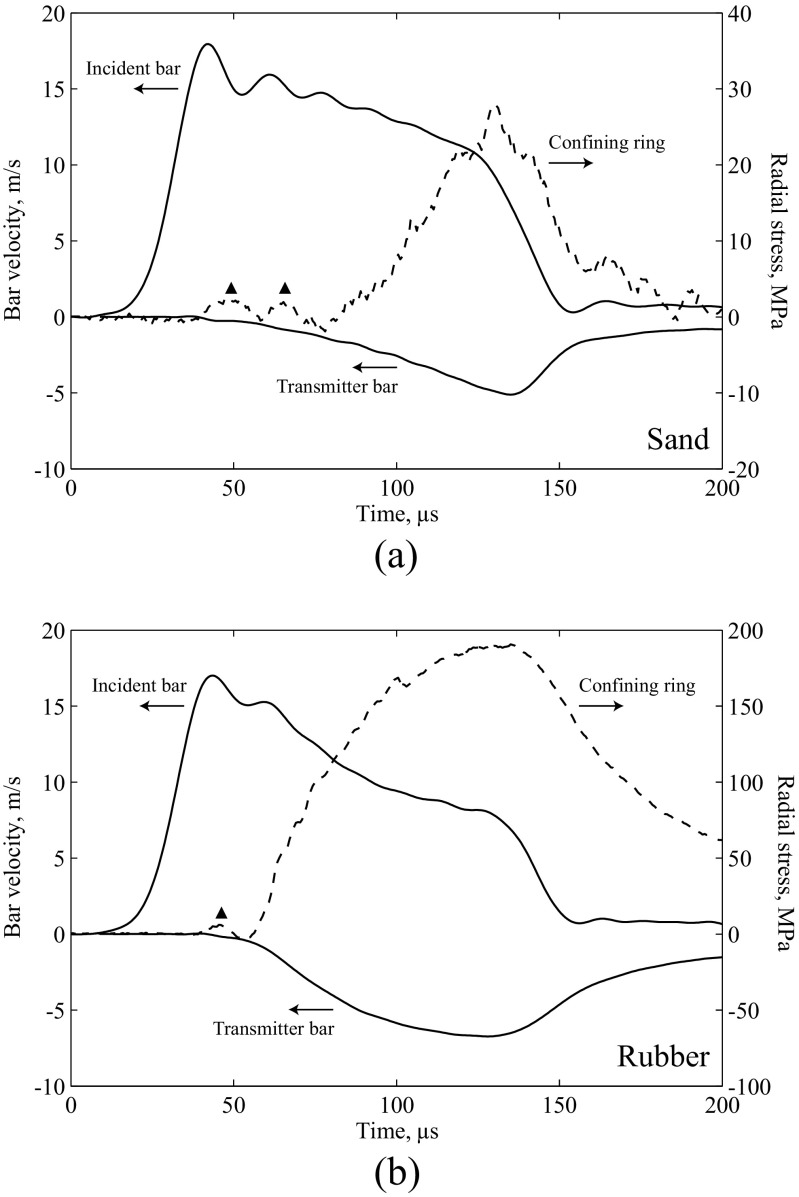
Relationship between incident and transmitter bar velocity and radial stress measurement in SHPB experiments on (**a**) 5 mm sand specimen, (**b**) 5 mm rubber specimen. Velocity positive towards the coil

The magnitude of the EMF generated in the strain gauge circuit is sensitive to the position of the gauge lead wires, and so it is not possible to post-process the data to remove the additional signals. Instead, the best approach in future experiments with radial strain measurements would be to use pressure bars which are non-ferromagnetic, removing the potential for magnetisation. If ferromagnetic bars are required for their mechanical properties, it may also be possible to align the gauge lead wires with the field around the bar to minimise interference. Use of a twisted pair of lead wires would also help to reduce the noise measured in the circuit by cancelling out the generated EMFs. Alternatively, a measurement method could be adopted which remains unaffected by any electromagnetic effects which are present. For example, interferometry could be used to measure the radial strain in the confining ring, and could be analysed in a similar manner to the circumferential strain gauge measurements to provide a recording of radial stress.

In summary, this investigation used an induction coil to identify the movement of magnetised pressure bars as the source of electromagnetic interference in measurements of radial stress during SHPB experiments. The potential contribution of sand particle breakage to this effect was also investigated, and deemed not to be significant. Recommendations were made to reduce the effects of electromagnetic interference and provide reliable radial stress measurements, which are crucial for the high-strain-rate characterisation of cohesionless materials such as soils.
